# The therapeutic effects of excimer laser coronary atherectomy therapy for in-stent restenosis chronic total occlusions

**DOI:** 10.1186/s12872-021-02208-x

**Published:** 2021-08-18

**Authors:** Hui Li, Hu Ai, Le Li, Naixin Zheng, Guodong Tang, Guojian Yang, Ying Zhao, Fucheng Sun, Huiping Zhang

**Affiliations:** 1 Peking University Fifth School of Clinical Medicine, Beijing, People’s Republic of China; 2 Cardiology Department, Beijing Hospital, National Center of Gerontology, Beijing, People’s Republic of China

**Keywords:** Excimer laser coronary atherectomy, Chronic total occlusion, In-stent restenosis

## Abstract

**Objectives:**

To evaluate the safety and efficacy of excimer laser coronary atherectomy (ELCA) in patients with in-stent restenosis chronic total occlusions (ISR CTOs).

**Background:**

ISR CTOs are a challenge in percutaneous coronary intervention (PCI). Although they can be treated by ELCA, limited data are available on the effects of ELCA treatment in these patients.

**Methods:**

Fifty-nine consecutive patients underwent PCI for ISR CTOs at Beijing Hospital between December 2017 and September 2020. According to whether or not ELCA was performed, they were divided into two groups. Quantitative coronary angiography (QCA) analyses were performed routinely, including measurement of the minimal lumen diameter and calculation of the percentage diameter stenosis. The procedural success rate, the frequency of peri-procedural complications, and the incidence rates of major adverse cardiac events (MACEs) over nine months were assessed. The primary endpoint in the study was the percentage diameter stenosis.

**Results:**

Procedure success was achieved in most patients in both groups (75.9%). Patients in the ELCA group exhibited a lower percentage diameter stenosis (24.5 ± 9.09 vs. 35.1 ± 18.6, p = 0.048) and a larger minimal lumen diameter (2.36 ± 0.29 mm vs. 1.78 ± 0.64 mm, p < 0.001) than those in the control group and the 9-month incidence rates of MACEs did not differ (9.5% vs 15.8%, p = 0.699).

**Conclusions:**

This study demonstrated that ELCA may be a safe and effective technique in the treatment of ISR CTOs, and the use of ELCA can achieve good immediate angiographic results, as measured by QCA, without increasing peri-procedural complications or the incidence rates of 9-month MACEs.

## Introduction

Stent therapy remains the first-line treatment for patients with severe coronary heart disease. Despite improvements in stent design, pharmacotherapy, and the polymers used in modern drug-eluting stents (DESs), approximately 5–10% of patients will experience in-stent restenosis (ISR) during angiographic follow-up [1, 2]. ISR continues to pose a therapeutic challenge and can be classified as focal, diffuse (with or without involvement of the stent edge), and chronic total occlusion (CTO) based on the results of angiography [3]. Due to the diffuse neointimal proliferation or neoatherosclerosis that may occur in the stents, the most severe ISR CTOs are more challenging to treat, and the outcomes of conventional treatments such as plain old balloon angioplasty (POBA), drug-coated balloon (DCB) angioplasty, and new stent implantations are usually unsatisfactory [4–6].

One treatment option is excimer laser coronary atherectomy (ELCA), which uses a xenon chloride laser generator to generate a 308 nm laser that can reduce plaque volume. ELCA has acceptable indications, including in the treatment of ISR lesions [7]. However, the outcomes of patients with ISR CTOs who have undergone ELCA treatment have not been previously described. Therefore, the aim of this study was to evaluate the therapeutic efficacy and safety of ELCA therapy in this population.

## Materials and methods

### Study population

We started a retrospective and descriptive study, the clinical and angiographic records of consecutive patients who underwent percutaneous coronary intervention (PCI) for ISR CTO by experienced surgeons at Beijing Hospital between December 2017 and September 2020 were reviewed. The indications to perform PCI were stable angina with viable myocardium and acute coronary syndrome. Data were collected and recorded in a dedicated CTO registry. The study protocols were approved by the ethics committee of Beijing Hospital, the study was conducted in accordance with the Declaration of Helsinki, and all patients signed informed consent to undergo coronary angiography and the intervention procedure.

The inclusion criteria were as follows: patients must have met the criteria for the diagnosis of ISR, any previous stent implantation must have occurred more than six months before the study, the coronary angiography must have revealed diffuse hyperplasia in the stent, and the diameter of the lumen must have been narrowed by more than 50% [8]. At the same time, the characteristics of the ISR lesions had to be consistent with the definition of CTO, the thrombolysis in myocardial infarction (TIMI) grade for forward blood flow must have been 0, and the estimated time of occlusion had to exceed three months [9]. Estimation of the duration of occlusion was based on the time from the first onset of angina, previous history of myocardial infarction (MI) in the target vessel territory, or comparison with the results of a prior angiogram.

### Procedure and devices

All of the angiographic images collected were archived in an electronic database; these images and the intervention procedures were reviewed for all patients. All procedures were performed via the radial or femoral route using a 6 F or 7 F sheath and guiding catheter (Medtronic, Santa Rosa, CA). Before the initiation of interventional therapy, all patients were treated with a loading dose of dual antiplatelet therapy that included acetylsalicylic acid 300 mg (Bayer, Berlin, German) combined with clopidogrel (Sanofi, Paris, France) 300 mg or ticagrelor (AstraZeneca, Cambridge, UK) 180 mg. The administration of intraoperative anticoagulation therapy consisting of unfractionated heparin or bivalirudin (Salubris, Shenzhen, China) was at the discretion of the operators. In patients who did receive such therapy, the dosage of unfractionated heparin was 70–100 IU/kg administered intravenously, and 1,000 units of unfractionated heparin were infused every hour. Bivalirudin was administered intravenously at a loading dose of 0.75 mg/kg, followed by continuous intravenous infusion at a dose of 1.75 mg/kg/h until 2 h after the procedure. For patients with chronic kidney disease, the dosage was adjusted according to the creatinine clearance rate.

Antegrade or retrograde technology was used to guide the wire through the occluded lesions. Patients in the non-ELCA group were treated with balloons to expand the lumen at the site of the lesion; subsequently, different sizes of DCBs or DESs were deployed according to the diameter of the blood vessel.

A CVX-300 excimer laser generator (Spectranetics, Colorado, Springs, USA) and select laser catheters were used in the ELCA group for ablation treatment. The diameter of the laser catheter was selected at the discretion of the operators according to the reference vessel and generally did not exceed half of the reference blood vessel’s diameter. During the laser ablation treatment, in strict accordance with the operating specifications, the advance speed of the laser catheter was controlled a rate of 1 mm/s, and the intravenous bolus of physiological saline was continuously injected at a speed of 1 mL/s. The bolus of contrast agent was avoided during the treatment. Following ELCA treatment and according to the angiography, different types of balloons were selected for further dilation. Most patients were treated with DCBs at the stented segment. For any coexisting proximal or distal lesions of the stents, new DESs were usually deployed.

### Laboratory and electrocardiographic analyses

For all patients, the concentrations of troponin, creatine kinase, and low-density lipoprotein cholesterol (LDL-C) were determined before and 6–24 h after PCI, with subsequent serial measurements performed in case there was any relevant biomarker elevation or patient complaints. Twelve-lead electrocardiographs were obtained before and after PCI, before hospital discharge, and upon suspicion of acute ischemia.

### Quantitative coronary angiography (QCA) analysis

QCA analysis was performed offline using Centricity Cardiology CA 1000 software version 2.0 (General Electric, USA). All measurements (baseline and final) were conducted according to the current standards, with the outer diameter of the contrast-filled catheter used as the calibration standard. The measurements included the length of the lesion before intervention, which consisted of the stented segment in addition to the 5 mm proximal and distal margins, the diameter of the reference vessel, the minimal lumen diameter (MLD), and the percentage diameter stenosis after the procedure. The percentage diameter stenosis was defined as follows:

$$\left( {\left[ {{\text{reference vessel diameter}}{-}{\text{MLD}}} \right]/{\text{reference vessel diameter}}} \right) \times {1}00\%$$

For the measurement of the MLD, an angiographic sequence without vascular constriction was selected. Two independent researchers measured the image sequence and took the average value for later analysis.

### Endpoints and definitions

The primary endpoint in the study was the percentage diameter stenosis, and the secondary endpoint was the 9-month incidence rates of major adverse cardiac events (MACEs), which were defined as events requiring hospitalization from MI, clinically driven target lesion revascularization (TLR) following the index PCI, and cardiac death. All clinical endpoints were defined according to the criteria of the Academic Research Consortium [10]. TLR was defined as any repeated percutaneous intervention or surgical bypass of the target lesion. Cardiac death was defined as death resulting from cardiovascular causes, including MI, heart failure, and sudden cardiac death, among others.

PCI-related MI was defined as type 4a MI according to the fourth universal definition of MI [11]. PCI-related MI occurring ≤ 48 h after the index procedure was arbitrarily defined as an elevation of cardiac troponin values > 5 times that of the 99^th^ percentile upper reference limit (URL) in patients with normal baseline values, or a rise in cardiac troponin levels > 20% of the baseline value when it is exceeded the URL, and at least one of the following criteria: new electrocardiographic changes indicative of ischemia, development of new pathological Q waves, angiographic findings consistent with a procedural flow-limiting complication, such as coronary dissection, occlusion of a major epicardial artery or graft, a thrombotic side-branch occlusion, disruption of collateral flow, or distal embolization. During the follow-up period, MI was defined as an increase in cardiac troponin levels above the 99th percentile URL and symptom recurrence, with or without new ST-segment changes.

Procedural success was defined as final percentage diameter stenosis ≤ 30% in the presence of grade 3 TIMI flow, without in-hospital death, emergency coronary artery bypass graft (CABG) surgery, and/or the need for repeat PCI during the index hospitalization period. The Japan CTO (J-CTO) score was calculated for each lesion based on the occlusion length, stump morphology, presence of calcification, presence of tortuosity, and any prior attempt to open up the CTO [12].

### Data management and clinical event adjudication

In-hospital adverse events were recorded until discharge from the hospital. The 9-month clinical follow-up data were obtained at visits to outpatient clinics or, if infeasible, by telephone follow-up and/or administration of a medical questionnaire.

### Statistical analyses

All statistical analyses were conducted using Statistical Package for the Social Sciences (SPSS) 23.0 software (SPSS Inc, Chicago, IL). Continuous variables with a normal distribution are presented as the mean ± SD, whereas those with a nonnormal distribution are presented as the median (interquartile range). Continuous variables were compared using independent-samples *t*-tests or nonparametric Mann–Whitney U tests. Categorical data are presented as counts (n) and percentages, and compared with chi-square or Fisher’s exact tests. All tests were two-tailed, and values less than 0.05 were considered statistically significant.

## Results

### Baseline clinical characteristics

**Fig. 1:**
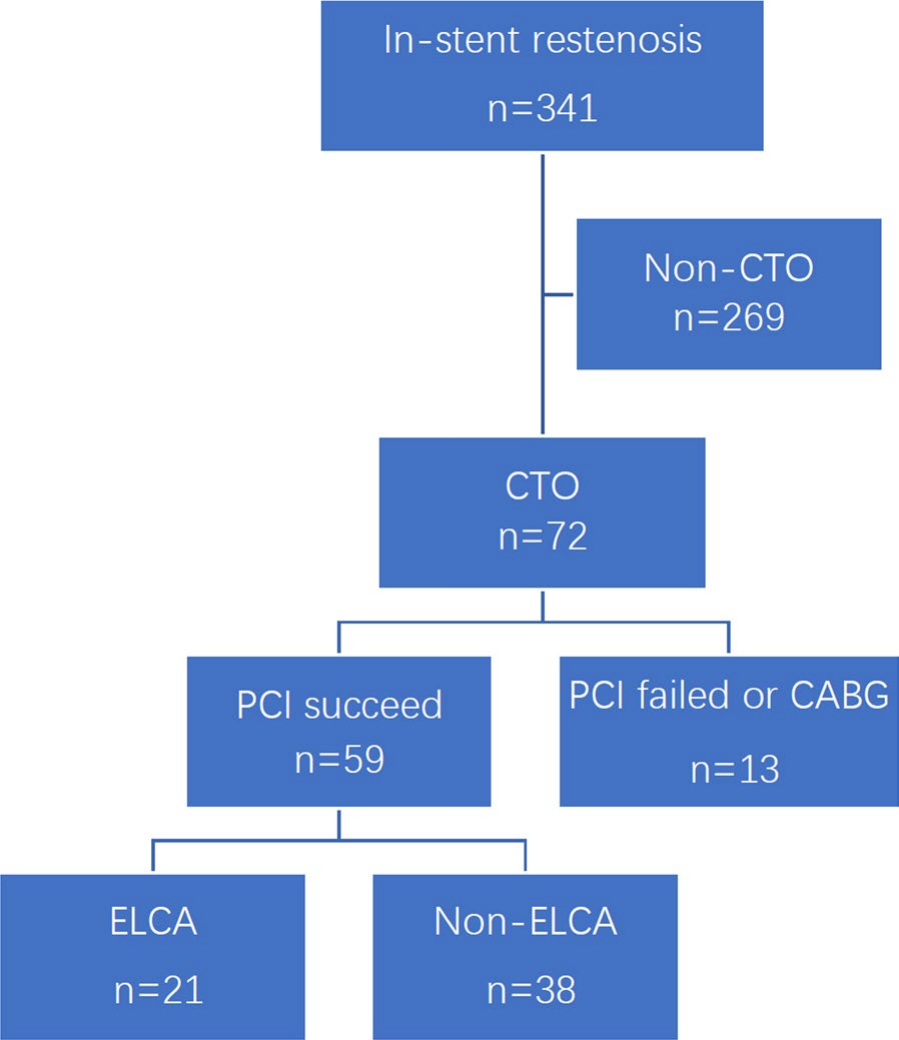
Flow chart of the study algorithm

A total of 341 patients were diagnosed with ISR by angiography at Beijing Hospital; of these patients, 72 had CTOs confirmed by angiographic images and medical history. A total of 59 patients received PCI. According to whether ELCA was performed or not, the patients were divided into two groups, the ELCA group and the non-ELCA group (Fig. 1).

There were no significant differences in the baseline clinical variables between the ELCA group and the non-ELCA group (Table 1). Of the study population, 83% were male, and 56% were diagnosed with a previous MI. Most patients exhibited a normal left ventricular ejection fraction (LVEF) and normal kidney function. A total of 39% of patients presented with acute coronary syndrome.

**Table 1:** Baseline clinical characteristics of the entire cohort

	Total n = 59	ELCA group n = 21	Non-ELCA group n = 38	*p value*
Age (years)	65.1 ± 10.1	65.5 ± 10.0	65.0 ± 10.3	0.850
Male sex (%)	49 (83.1)	17 (81.0)	32 (84.2)	0.733
Hypertension (%)	40 (67.8)	14 (66.7)	26 (68.4)	0.890
Hyperlipidemia (%)	37 (62.7)	14 (66.7)	23 (60.5)	0.641
Diabetes mellitus (%)	29 (49.2)	12 (57.1)	17 (44.7)	0.361
Previous MI (%)	33 (56.3)	11 (52.4)	22 (57.9)	0.683
Previous CVA (%)	10 (16.9)	6 (28.6)	4 (10.5)	0.144
Current smoker (%)	34 (57.6)	15 (71.4)	19 (50.0)	0.111
eGFR (mL/min/1.73 m^2^)	90.6 ± 28.6	98.4 ± 26.3	88.5 ± 29.2	0.341
LVEF %	59.5 (49.3, 60.0)	49 (45, 63)	60 (52, 60)	0.627
LDL-C (mmol/L)	2.03 ± 0.68	2.56 ± 1.00	1.97 ± 0.57	0.244
Clinical presentation				
Stable angina (%)	36 (61.0)	14 (66.7)	22 (57.9)	0.508
ACS (%)	23 (39.0)	7 (33.3)	16 (42.1)	0.508

Data are presented as n (%) or mean ± standard deviation or median (interquartile range)

MI , myocardial infarction; CVA , cerebrovascular accident; eGFR , estimated glomerular filtration rate; LVEF , left ventricular ejection fraction; LDL-C , low density lipoprotein-cholesterol; ACS , acute coronary syndrome

### Angiographic and procedural characteristics

**Fig. 2:**
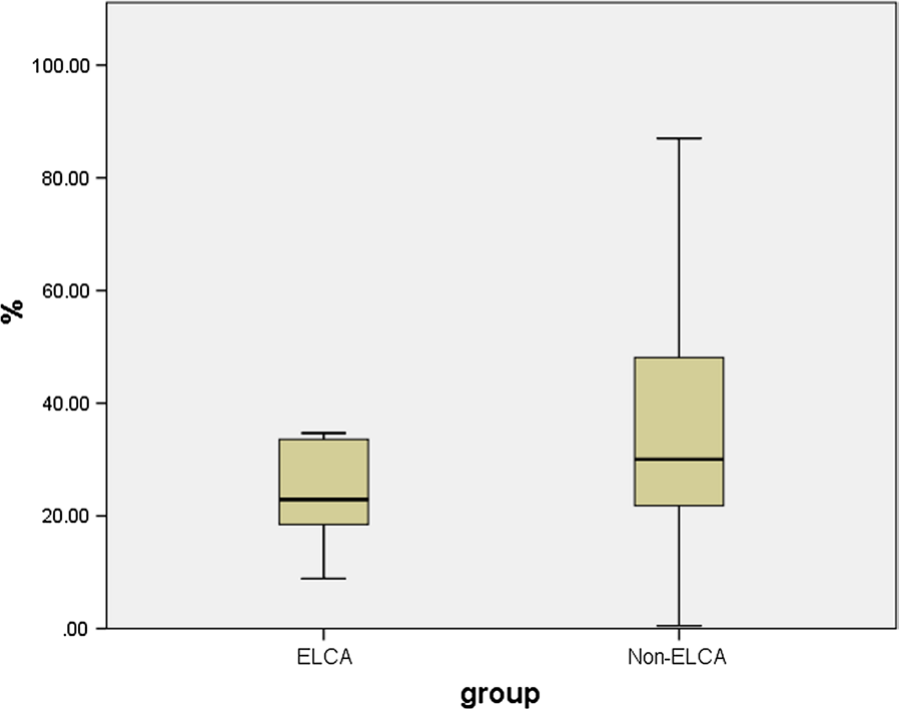
The percentage diameter stenosis of the final angiography

**Fig. 3:**
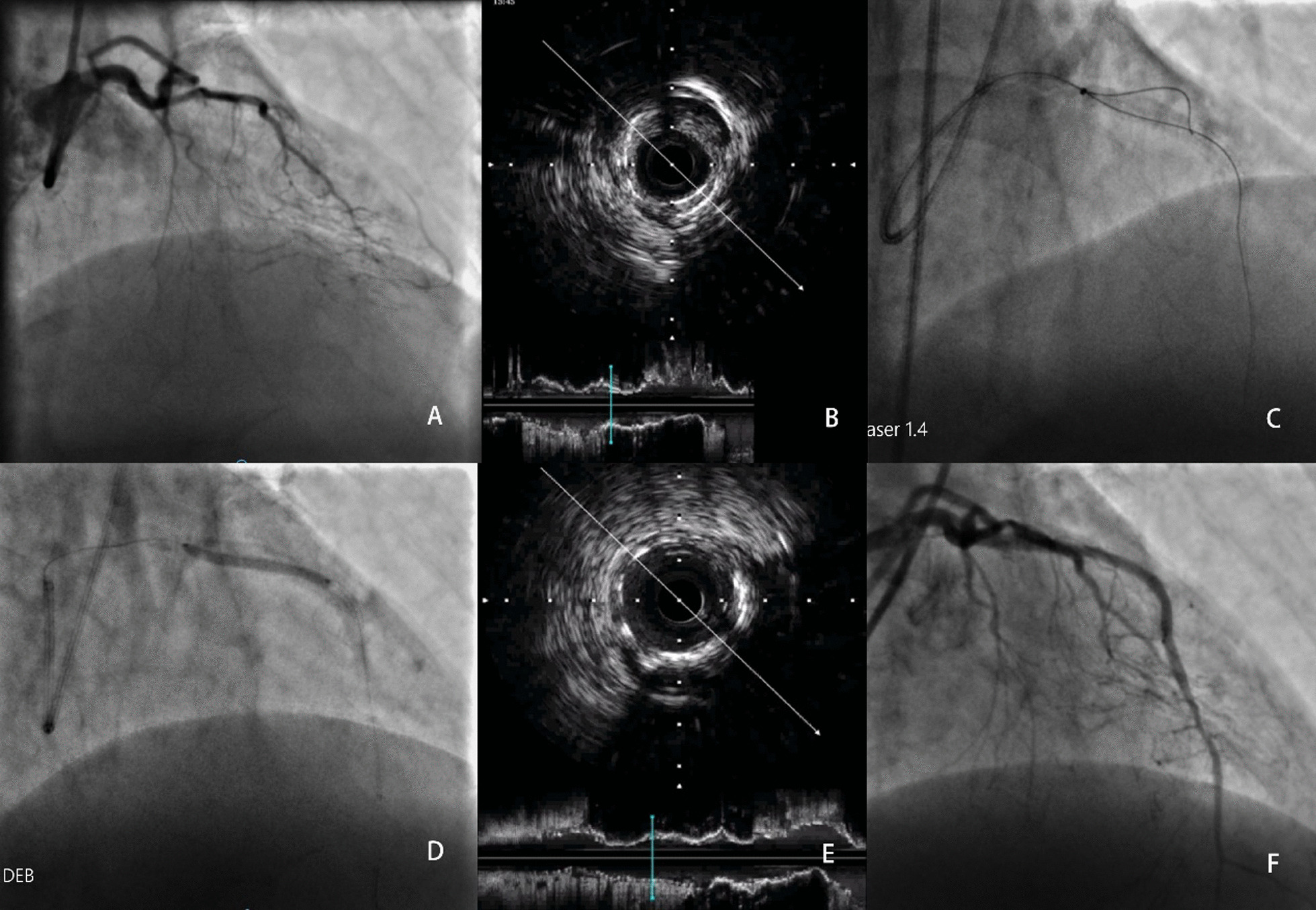
A 55-year-old patient treated via ELCA and DCB. **a** Development of in-stent restenosis chronic total occlusion in the left anterior descending artery. **b** IVUS showing diffuse hyperplastic tissue in the stent segment. **c** ELCA conducted with a 1.4 mm catheter. **d** A 3.0–30 mm DCB deployed in the previous stent. **e** IVUS demonstrating the presence of mild residual hyperplastic tissue in the stent segment and lumen area. **f** The final angiography of the artery post-procedure. ELCA = excimer laser coronary atherectomy; IVUS = intravascular ultrasound; DCB = drug-coated balloon

The angiographic and procedural characteristics of the two groups are summarized in Table 2. The ELCA group had a higher mean J-CTO score than the non-ELCA group (2.57 ± 0.79 vs. 1.67 ± 0.70, respectively; p = 0.007), although the syntax score did not differ between groups (p = 0.141). Approximately 80% of the patients were treated with DCBs, although more DESs were used in the ELCA group than in the non-ELCA group (61.9% vs. 34.2%, respectively; p = 0.040). No significant differences were noted between groups in terms of the lesion location or lesion length, or the reference lumen diameter. DESs were used in 44.1% of the patients, although none of the stents were deployed in the previous stent segments. The ELCA group had a higher procedural success rate in terms of raw percentages, but there were no significant differences between groups. The percentage diameter stenosis was lower in the ELCA group than in the non-ELCA group (Fig. 2), whereas the MLD after treatment was larger. A typical patient who underwent ELCA is shown in Fig. 3.

**Table 2:** Angiographic and procedural characteristics of the patients

	Total n = 59	ELCA group n = 21	Non-ELCA group n = 38	*p value*
Lesion location				
Left anterior descending (%)	27 (45.8)	14 (66.7)	13 (34.2)	
Left circumflex (%)	10 (16.9)	2 (9.5)	8 (21.1)	
Right coronary artery (%)	22 (37.3)	5 (23.8)	17 (44.7)	0.056
Multiple vessel disease (%)	55 (93.2)	20 (95.2)	35 (92.1)	1.000
Syntax score	19.4 ± 7.6	21.9 ± 3.3	18.7 ± 8.3	0.141
J-CTO score	1.87 ± 0.81	2.57 ± 0.79	1.67 ± 0.70	0.007
Treatment with DCB (%)	49 (83.1)	16 (76.2)	33 (86.8)	0.306
Treatment with DES (%)	26 (44.1)	13 (61.9)	13 (34.2)	0.040
IVUS	12 (20.3)	5 (23.8)	7 (18.4)	0.739
OCT	1 (1.7)	1 (4.8)	0 (0)	0.356
Before intervention				
Lesion length (mm)	33.5 ± 11.3	36.4 ± 13.2	32.7 ± 10.7	0.363
Reference lumen diameter (mm)	2.79 ± 0.54	3.08 ± 0.52	2.72 ± 0.52	0.059
After intervention				
Minimum lumen diameter (mm)	1.90 ± 0.63	2.36 ± 0.29	1.78 ± 0.64	< 0.001
Percentage diameter stenosis	32.9 ± 17.5	24.5 ± 9.09	35.1 ± 18.6	0.048
Procedure success (%)	44 (75.9)	18 (85.7)	26 (70.3)	0.187
Fluoroscopic time (min)	37.3 ± 20.9	30.4 ± 16.4	39.2 ± 21.7	0.400
Contrast volume (mL)	230.4 ± 58.4	254.2 ± 52.6	223.7 ± 58.8	0.146

Data are presented as n (%) or mean ± standard deviation or the median (interquartile range)

J-CTO score: Japan chronic total occlusion score; DCB: drug-coated balloon; DES: drug-eluting stent; IVUS: intravascular ultrasound; OCT: optical coherence tomography; PCI: percutaneous coronary intervention; MI: myocardial infarction

### Peri-procedural complications and 9-month MACE outcomes

The incidence of each peri-procedural complication is documented in Table 3. All procedures were conducted without the occurrence of major adverse events, including death, subacute closure, and target lesion revascularization (TLR) during the hospitalization period. However, coronary perforation did occur in two patients in the ELCA group, the classifications of which were Ellis class 2, which are defined as pericardial or myocardial blushing in the absence of an exit hole exceeding 1 mm in size [13] and no occurrence of tamponade requiring pericardiocentesis. For coronary slow flow/ no re-flow complications that usually cause MI, the total peri-procedure complications did not exceed the sum of the individual complications. No cardiac death occurred, and no definite or probable stent thrombosis was recorded in any patient. A total of seven patients underwent TLR for binary restenosis, with no significant differences between both groups.

**Table 3:** Incidence of peri-procedural complications and 9-month MACEs

	Total n = 59	ELCA group n = 21	Non-ELCA group n = 38	p value
Peri-procedural complications	9 (15.3)	5 (23.8)	4 (10.5)	0.258
Coronary perforation	2 (3.4)	2 (9.5)	0	0.123
Coronary slow flow/no flow	4 (6.6)	2 (9.5)	2 (5.3)	0.812
PCI-related MI (%)	7 (11.9)	3 (14.3)	4 (10.5)	0.691
9-month MACEs	8 (13.6)	2 (9.5)	6 (15.8)	0.699
MI	1 (1.7)	0	1 (2.6)	1.000
TLR	7 (11.9)	2 (9.5)	5 (13.2)	0.732
Cardiac death	0	0	0	NA
Stent thrombosis	0	0	0	NA

Data are presented as n (%)

PCI, percutaneous coronary intervention; MI, myocardial infarction; MACEs, major adverse cardiac events; TLR, target lesion revascularization

## Discussion

The aim of the present study was to evaluate the immediate effects and 9-month outcomes of ELCA treatment in patients with ISR CTO. The main findings were as follows: (1) ELCA achieved good immediate angiographic results, as measured by QCA, without increasing the number of peri-procedural complications, including coronary perforation, coronary slow flow/no re-flow problems, and PCI-related MI. (2) ELCA did not alter the incidence of 9-month MACEs.

PCI with DESs is the current standard of care for the treatment of native coronary artery stenoses. However, the optimum percutaneous treatment strategy for patients presenting with ISR is still debated. Current studies have shown that for ISR, the use of DCBs can reduce the incidence of binary restenosis compared with that of POBA treatment [14]. DCBs offer the advantage of not requiring the implantation of an additional metallic layer when treating ISR, and they are recommended by the European Society of Cardiology and Chinese guidelines as the first-line treatment option [15, 16]. Because DCB technology has not yet received U.S. Food and Drug Administration approval for use in the coronary vasculature, repeated stent implantation and POBA are the regular choices in the USA [17].

Regardless of whether DCBs or DESs are used, it is impossible to truly remove the hyperplastic tissue or neoatherosclerosis present in the stent area. Unlike de novo lesions, this atherosclerotic plaque can expand and become redistributed. Due to the limitations of the original metallic stent structure, it is difficult to achieve sufficient expansion of ISR lesions through POBA. This inability to open up the coronary lumen reduces the therapeutic effects of the treatment. Therefore, it is common for a patient to experience binary restenosis within a short time following a procedure. When POBA is performed alone, the incidence of TLR at six months post-procedure is as high as 63.1% [18]. Lee et al. compared ISR CTOs and de novo CTOs that were mostly treated with DESs (> 90%); although the procedure success rate for treating ISR CTOs was higher because the previous stents could be used as a roadmap, the prognosis was worse, and the incidence of TLR was significantly higher (14% vs 4.3%, respectively; p < 0.001) [19]. Therefore, in this type of situation, techniques such as ELCA for removing the hyperplastic tissue could be a promising therapeutic option.

ELCA mainly exerts photothermal, photochemical, and photomechanical effects that result in the removal of hyperplastic tissue. Both in vitro and in vivo experiments have revealed that ELCA treatment can break the chemical bonds of cellular molecules by forming microbubbles in the cells. This can completely remove the proliferated cells by reducing them into microparticles, resulting in a therapeutic effect through plaque volume reduction [7, 20]. Another possible mechanism that could explain the benefits of performing ELCA for ISR is that ISR develops as a result of stent under-expansion, as the stent is constrained by external layers of calcium. ELCA can ablate the tissue around the stent and improve its ability to expand. At baseline, all of the patients in this study were experiencing CTO; therefore, the percentage diameter stenosis may be the best indicator of the effects of PCI. In this study, the patients treated by ELCA achieved a lower percentage diameter stenosis, which reflected a “debulking effect” of the treatment. Furthermore, the patients in the ELCA group had a larger reference lumen diameter than that of the non-ELCA group, which indicated that those with greater hyperplastic tissue burden can achieve a larger MLD (2.36 ± 0.29 mm vs. 1.78 ± 0.64 mm, respectively; p < 0.001).

The incidence rates of 9-month MACEs were similar in both groups, and most events were TLRs. The TLR rate was lower in the ELCA group than in the non-ELCA group (9.5% vs. 13.2%, respectively), although the groups did not significantly differ. Early clinical studies have shown that for diffuse in-stent restenosis, ELCA combined with POBA can achieve satisfactory therapeutic effects similar to the outcome of rotational atherectomy combined with POBA, although the TLR rate at one year post-procedure remained as high as 26% [21]. Herein, ELCA combined with DCB treatment for ISR CTOs resulted in a lower 9-month incidence rate of TLRs. However, due to an insufficient sample size, it was not possible to fully confirm the advantages of DEBs over POBA.

ELCA treatment can theoretically increase the total operation time; however, there were no significant differences in the fluoroscopic times or doses of contrast agents administered between the two groups. These two indicators were used instead of the overall operation time because these factors better reflect the extent of each patient’s injury and the complexity of the procedures. Some possible reasons for this were considered. For example, ELCA treatment itself does not require much time, generally speaking; once the wire passes the CTO lesion, the entire procedure can be completed in approximately 15 min, and ELCA treatment can reduce the amount of hyperplastic tissue in the area, which could reduce the subsequent balloon pre-dilation time.

In the present study, two patients (9.5%) in the ELCA group developed coronary perforation without clinical sequelae; however, coronary perforation is one of the recognized serious complications of PCI. Danek et al. [22], in a study based on data from the PROGRESS-CTO registry, the rate of coronary perforation was much higher when ELCA was used than when it was not (7% vs. 2%, respectively; p < 0.009). Protty et al. [23] also reported that ELCA was associated with a higher coronary perforation rate (odds ratio: 2.18, 95% confidence interval: 1.44–3.30) based on data from the British Cardiovascular Intervention Society database. For ISR CTO procedures, the wires may not remain in the true lumen as they are advanced in some segments; therefore, ELCA requires experienced operators, and operators must be cautious to avoid this kind of serious complication. Careful observation is recommended when a perforation does occur, as complications may develop over the course of several hours post-procedure. Laser light combined with simultaneous contrast injection can generate a powerful acoustic-mechanical effect, forming microbubbles that will increase the disruptive forces that result in an improved outcome, especially in cases of under-expanded stents [24, 25]. However, this is not our recommendation, as it could result in a higher perforation rate and more serious types of perforations.

### Study limitations

Some limitations of the present study should be noted. First, the study was retrospective in nature, so although the baseline clinical characteristics of the two groups completely matched, there may be some confounding factors that were not considered. Second, the angiographic images were analyzed by QCA analysis instead of more precise intracoronary imaging, such as IVUS or optical coherence tomography; this was because less than 30% of the included patients had those techniques applied during the intracoronary imaging. Third, this study is based on a small sample from a single center with a short time follow-up period; therefore, future studies with larger sample sizes and assessments of long-term prognosis are warranted. Fourth, the study population was strictly selected; thus, the results should be extrapolated with caution. Fifth, an undeniable learning curve was present when performing ELCA across subsequent cases. The strategy and technique for ISR CTOs conducted in a single center might not adequately reflect the outcomes of patients treated in other institutions. Finally, routine angiographic follow-up in patients who underwent PCI was not absolutely required in daily practice, which might have potentially induced a bias that affected the TLR rate.

## Conclusions

The results of this study demonstrated that ELCA may be an effective treatment for ISR CTOs. It can significantly improve the immediate angiographic effects without increasing the occurrence of peri-procedural complications or the incidence rates of 9-month MACEs.

## Data Availability

All data analyzed during the current study are available from the corresponding author upon reasonable request.

## Abbreviations

ACS: Acute coronary syndrome
CABG: Coronary artery bypass graft
CTO: Chronic total occlusion
CVA: Cerebrovascular accident
DCB: Drug-coated balloon
DES: Drug-eluting stent
eGFR: Estimated glomerular filtration rate
ELCA: Excimer laser coronary atherectomy
ISR: In-stent restenosis
IVUS: Intravascular ultrasound
LDL-C: Low-density lipoprotein cholesterol
LVEF: Left ventricular ejection fraction
MACE: Major adverse cardiac event
MI: Myocardial infarction
MLD: Minimal lumen diameter
OCT: Optical coherence tomography
PCI: Percutaneous coronary intervention
POBA: Plain old balloon angioplasty
QCA: Quantitative coronary angiography
TIMI: Thrombolysis in myocardial infarction
TLR: Target lesion revascularization
URL: Upper reference limit
